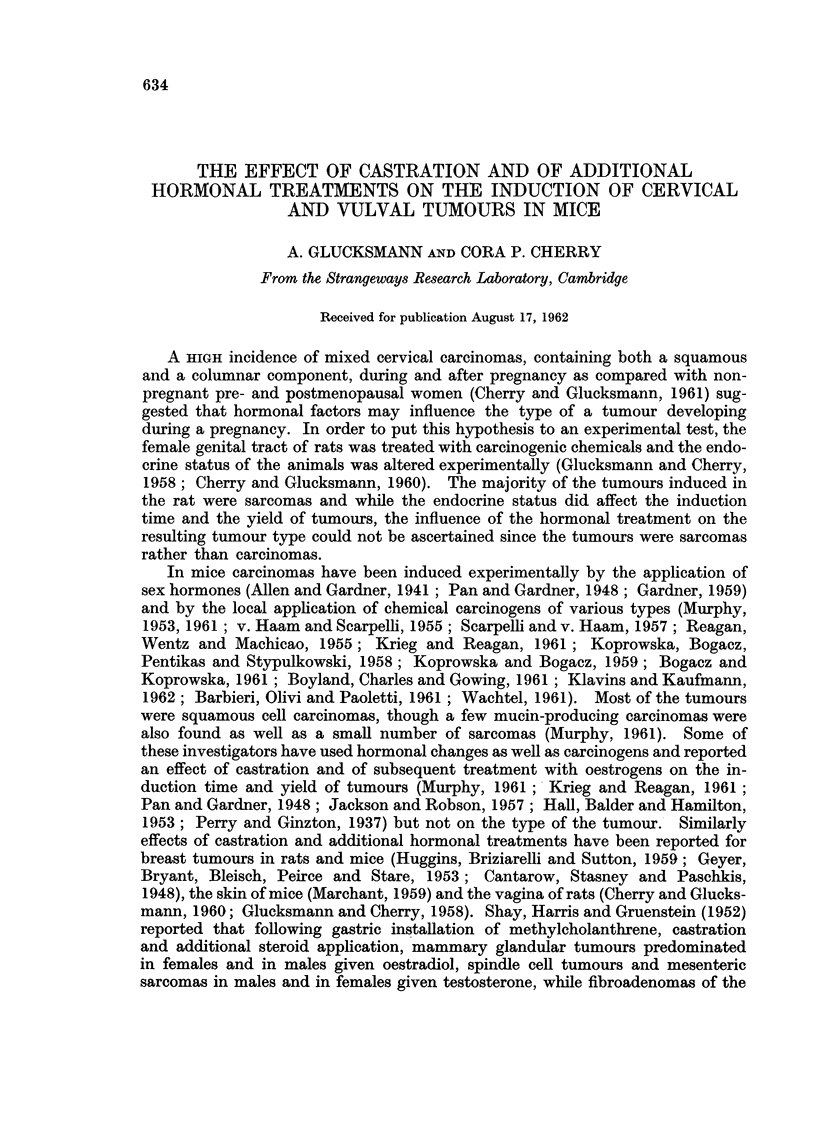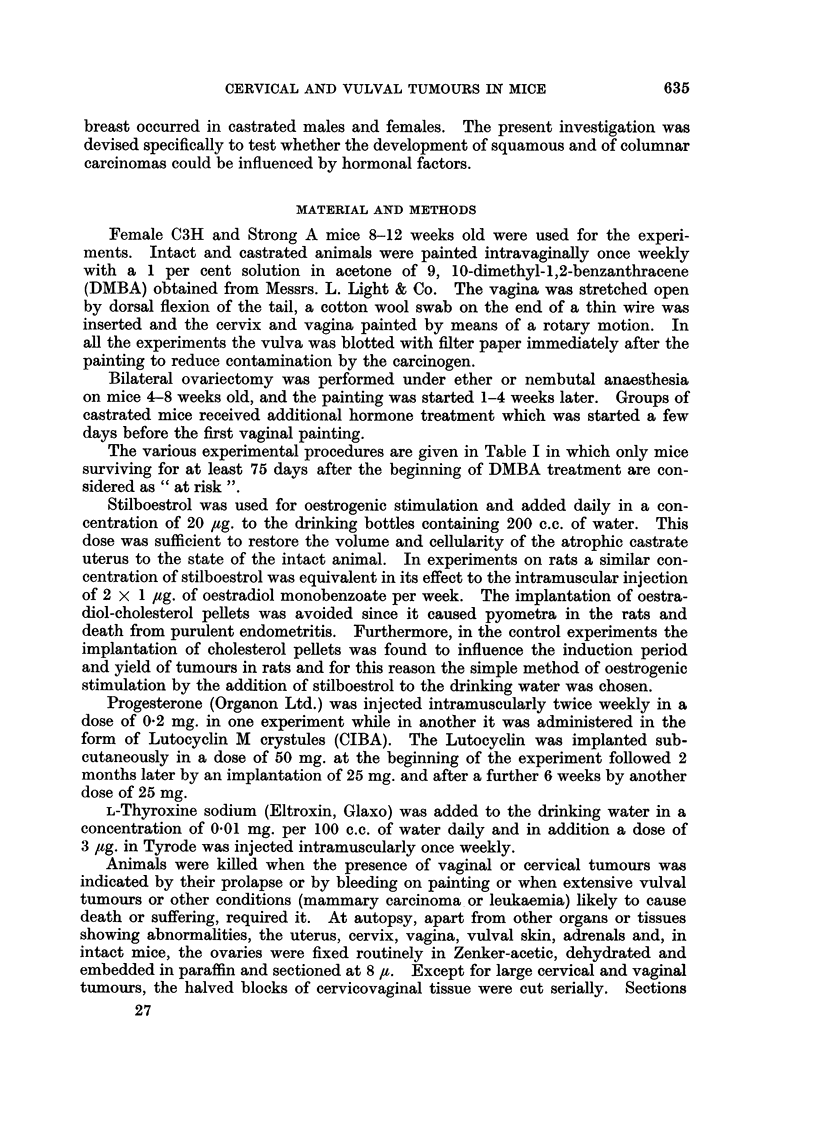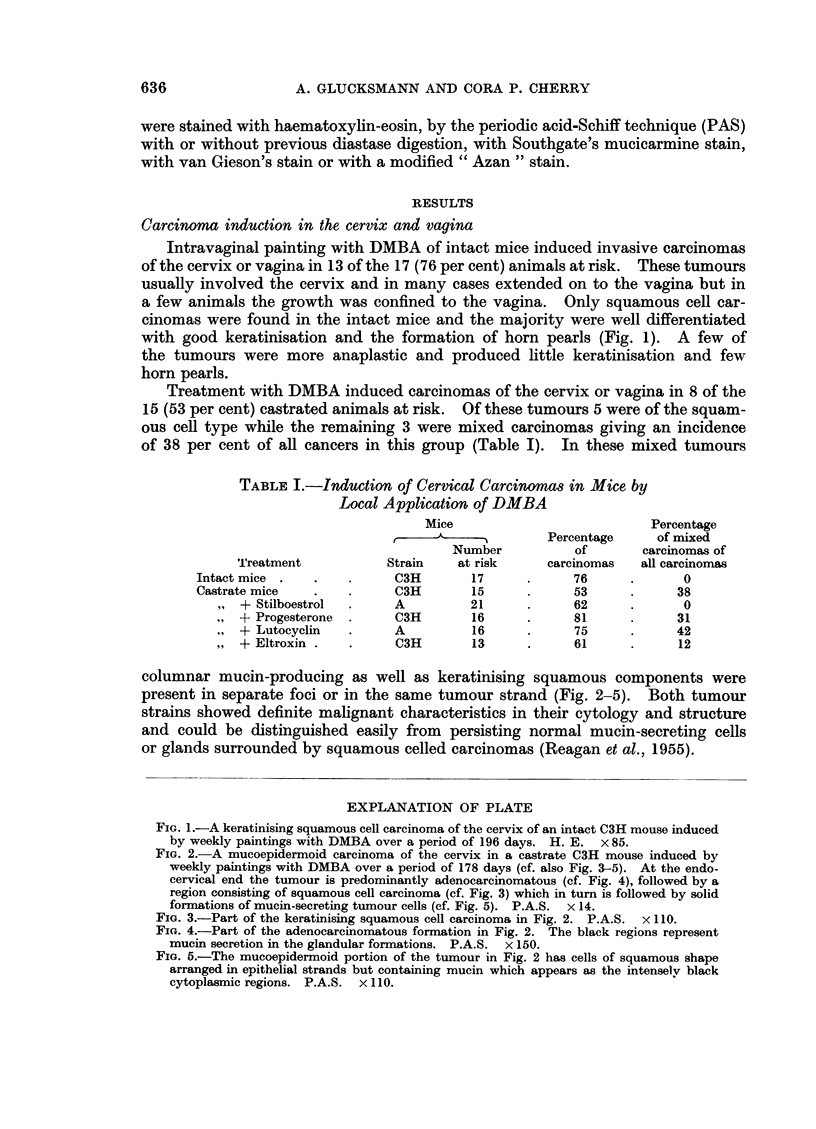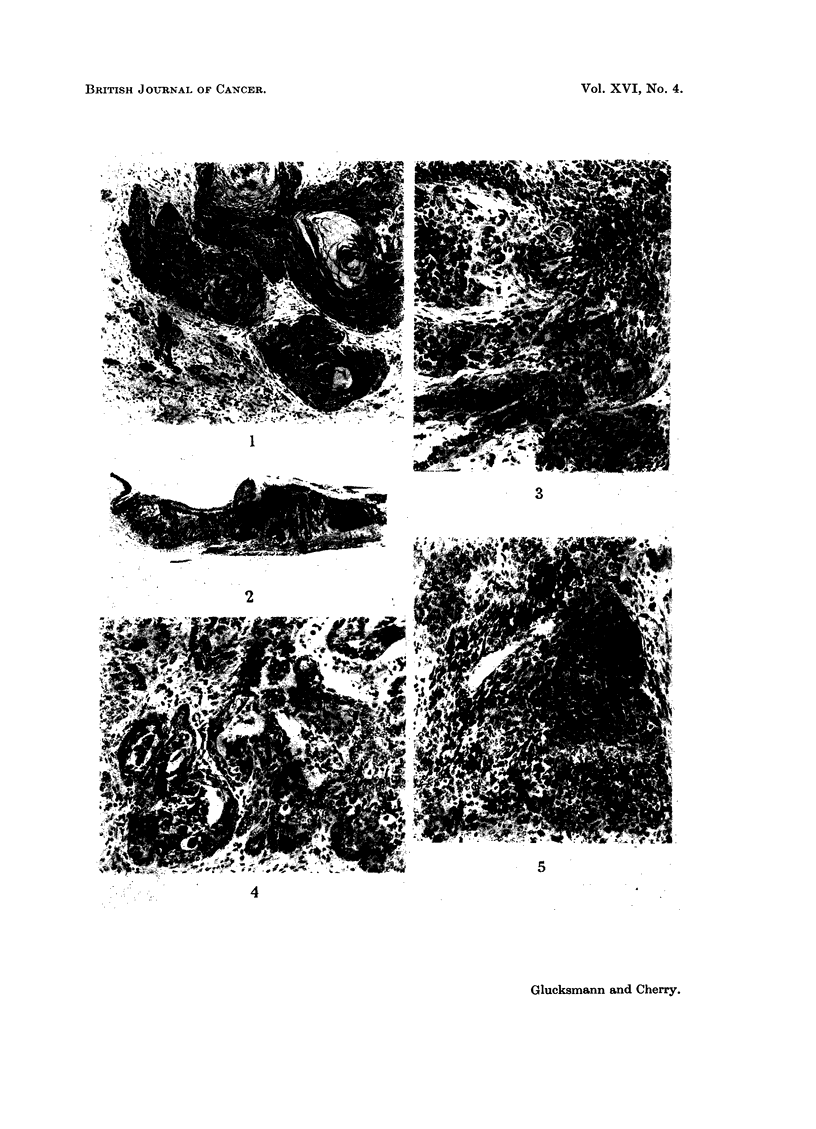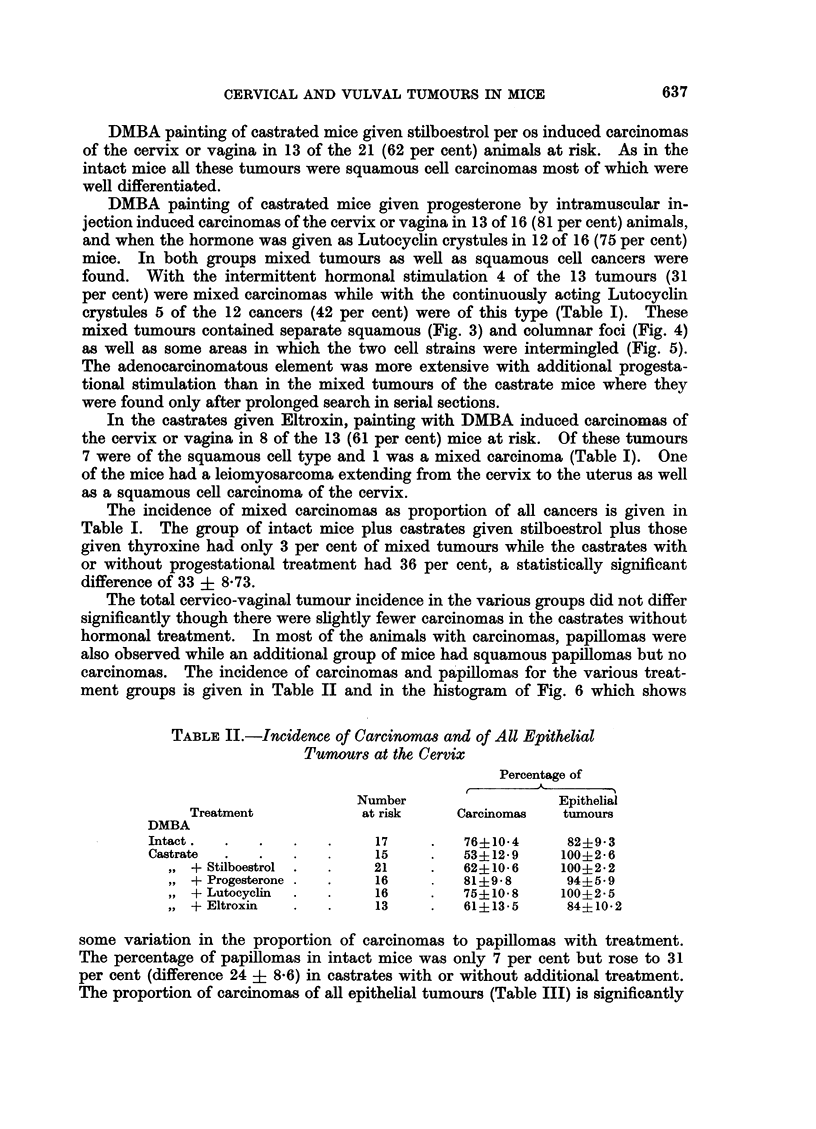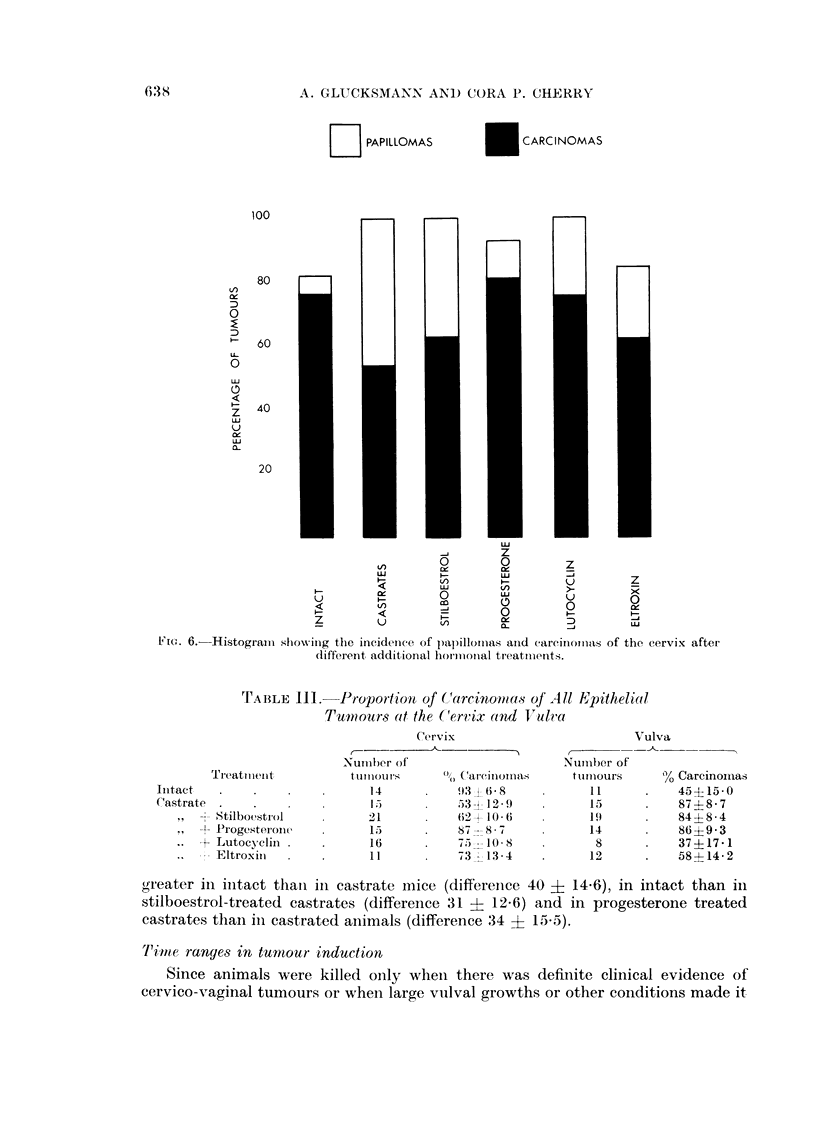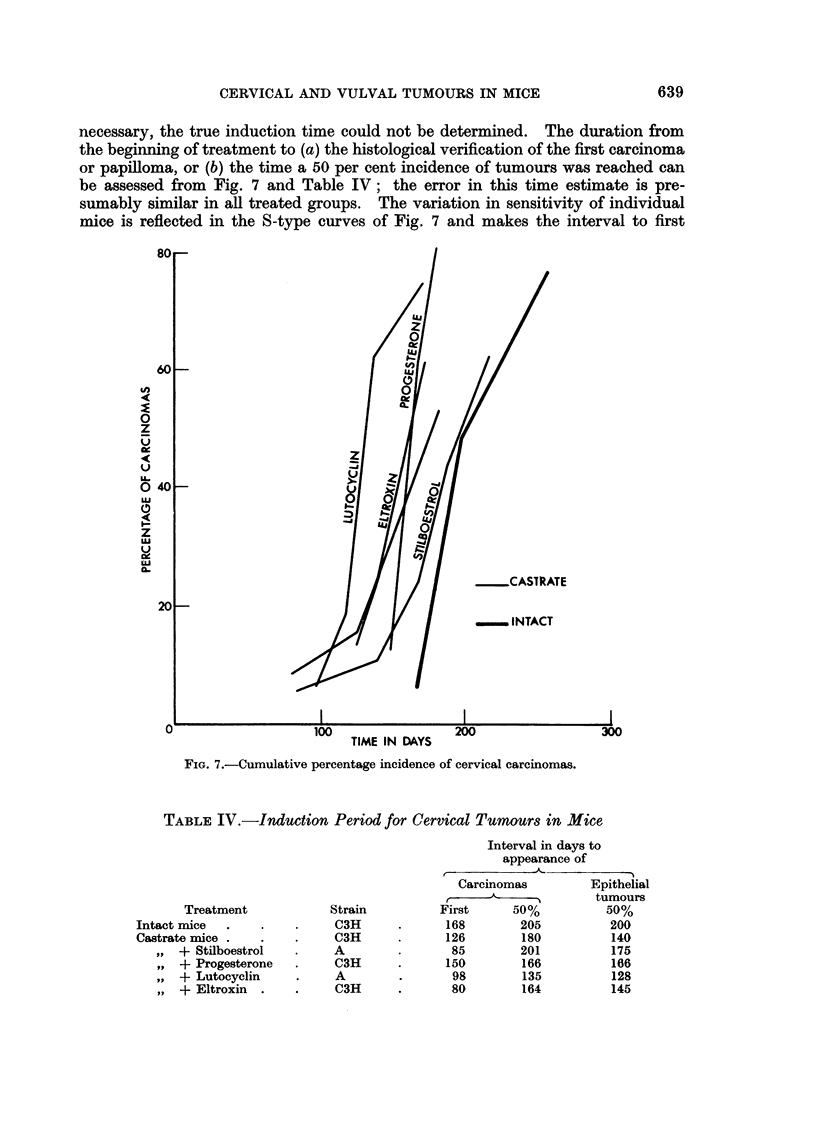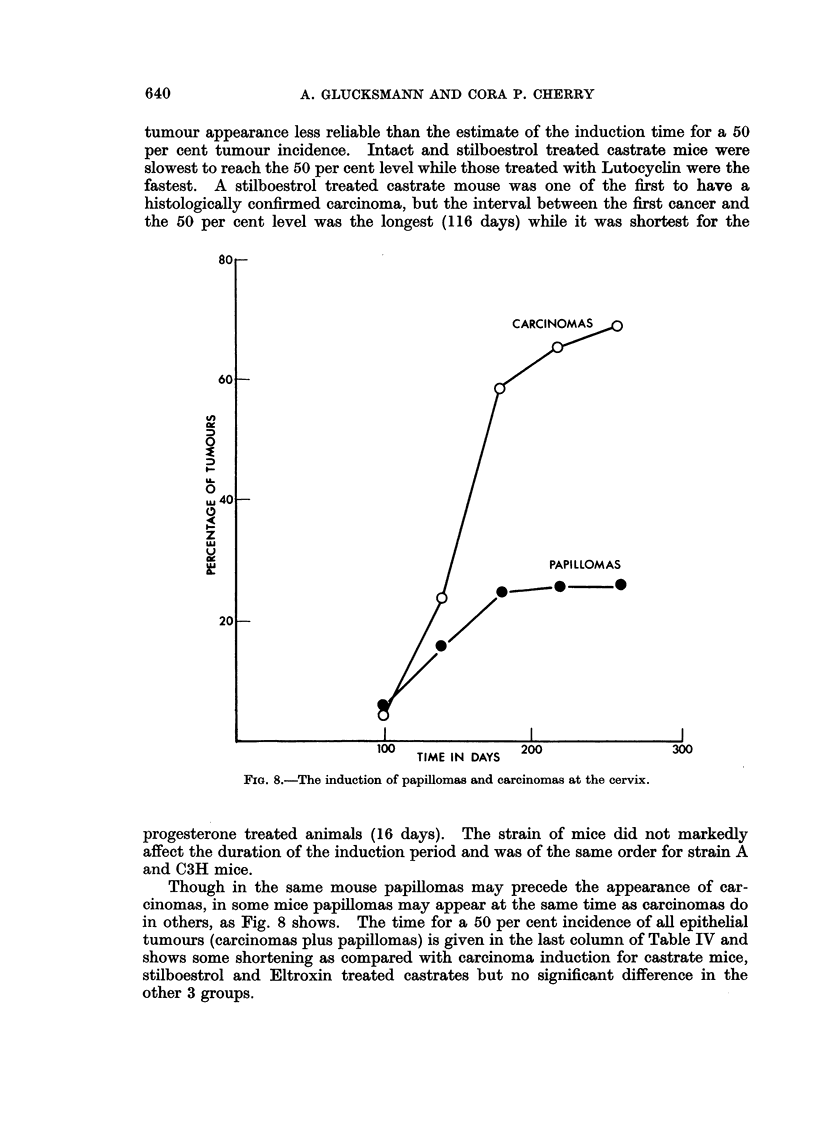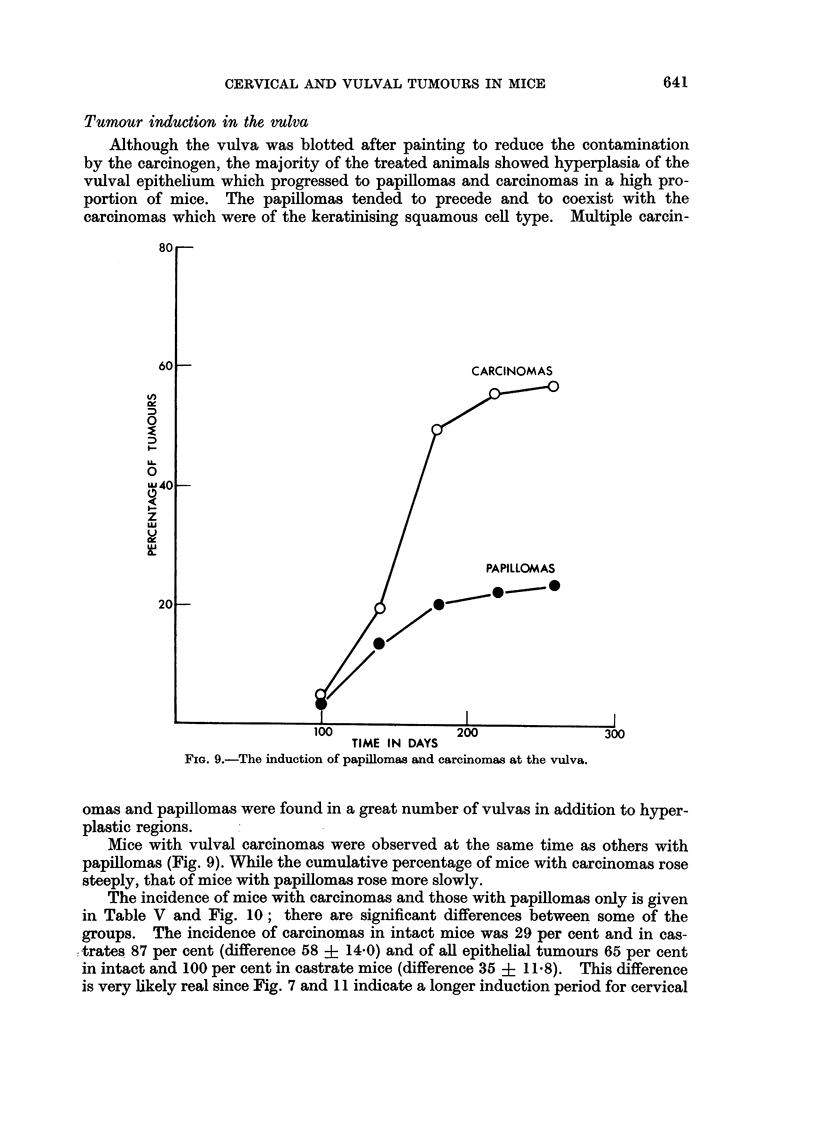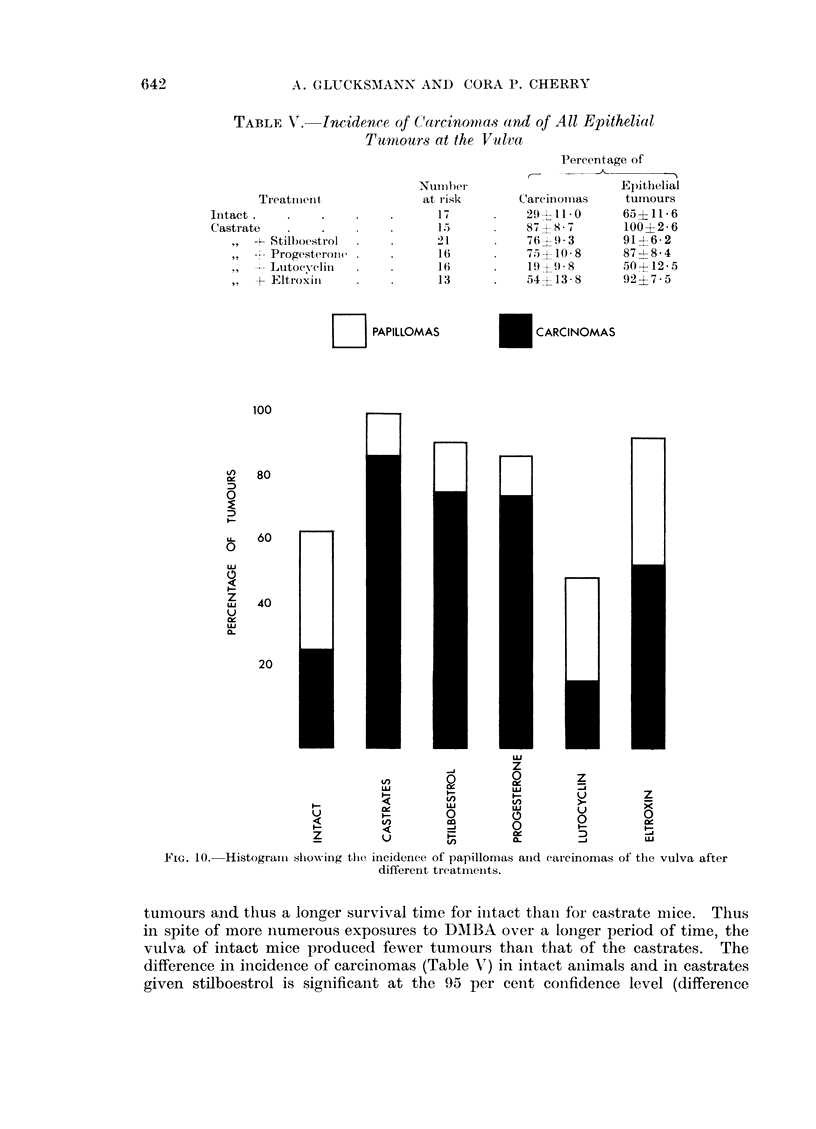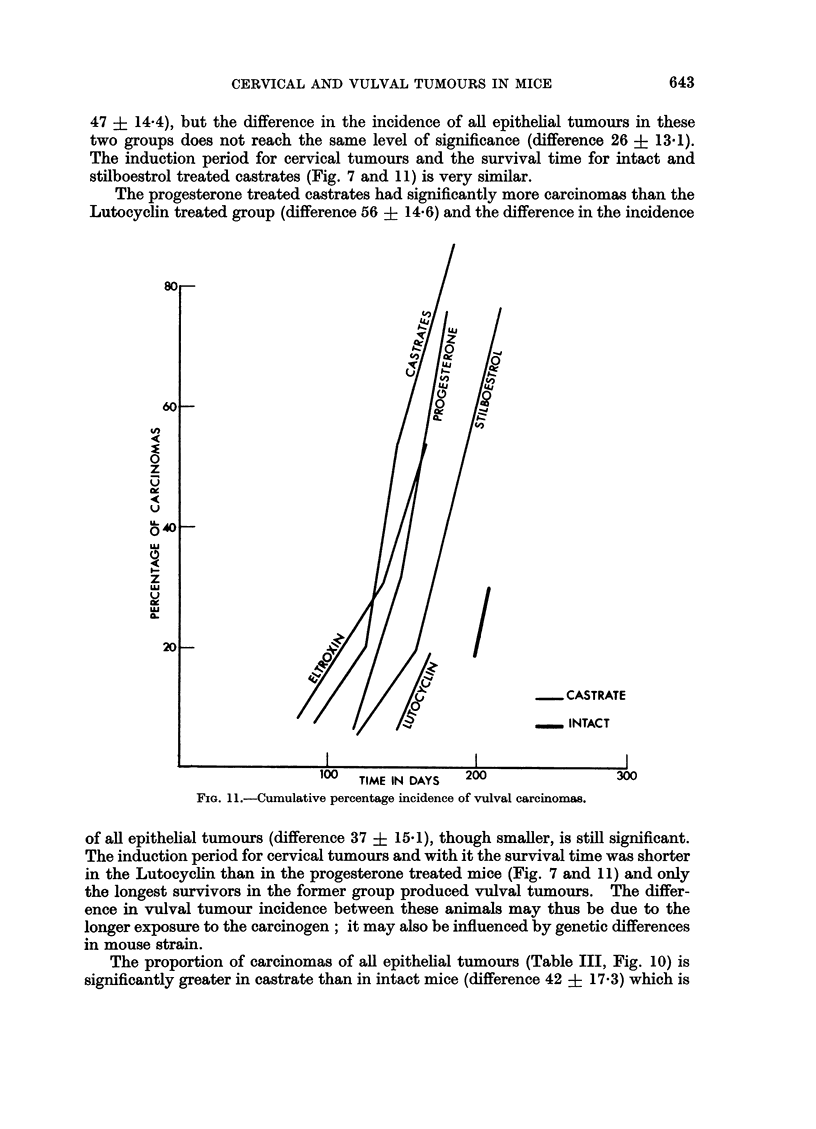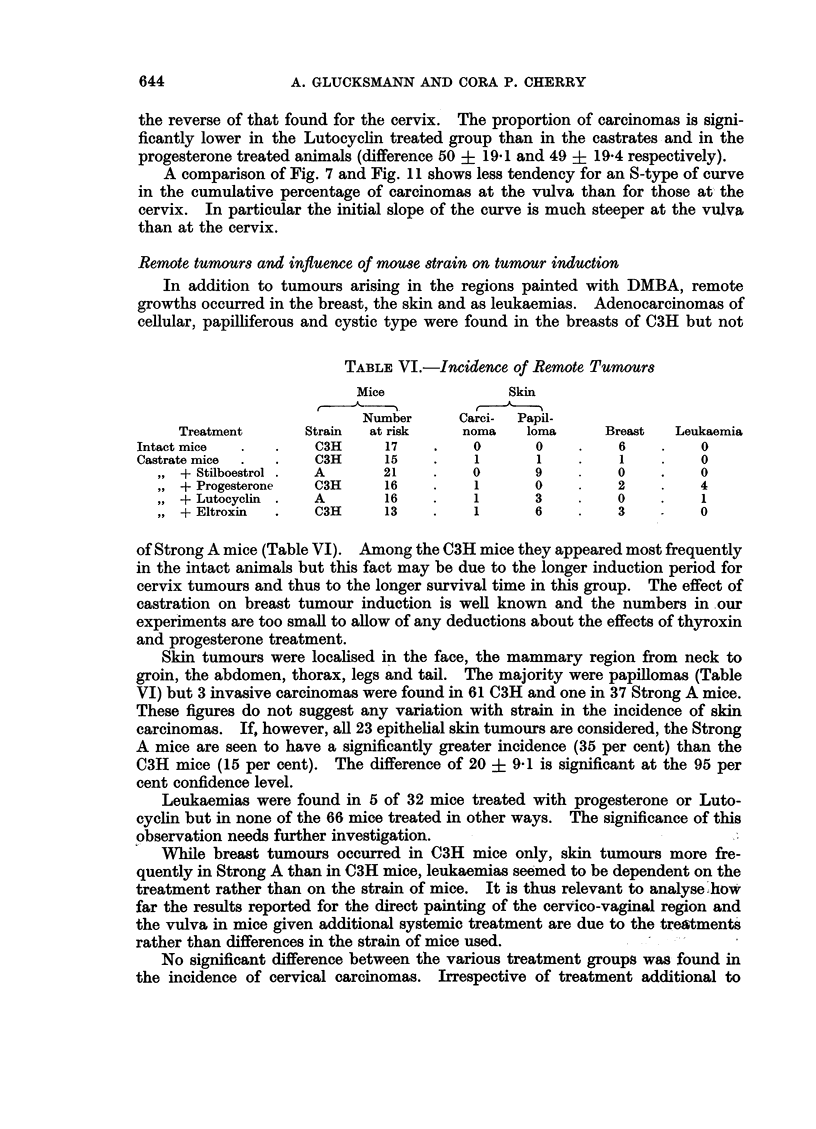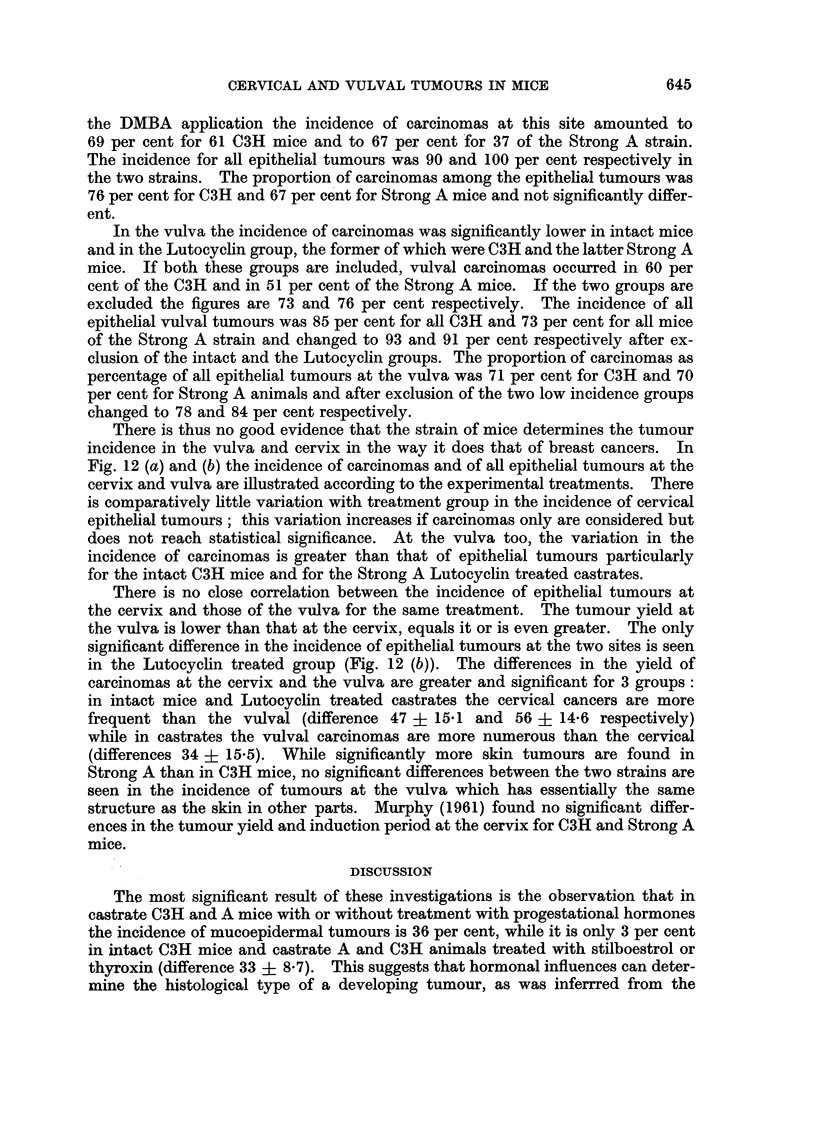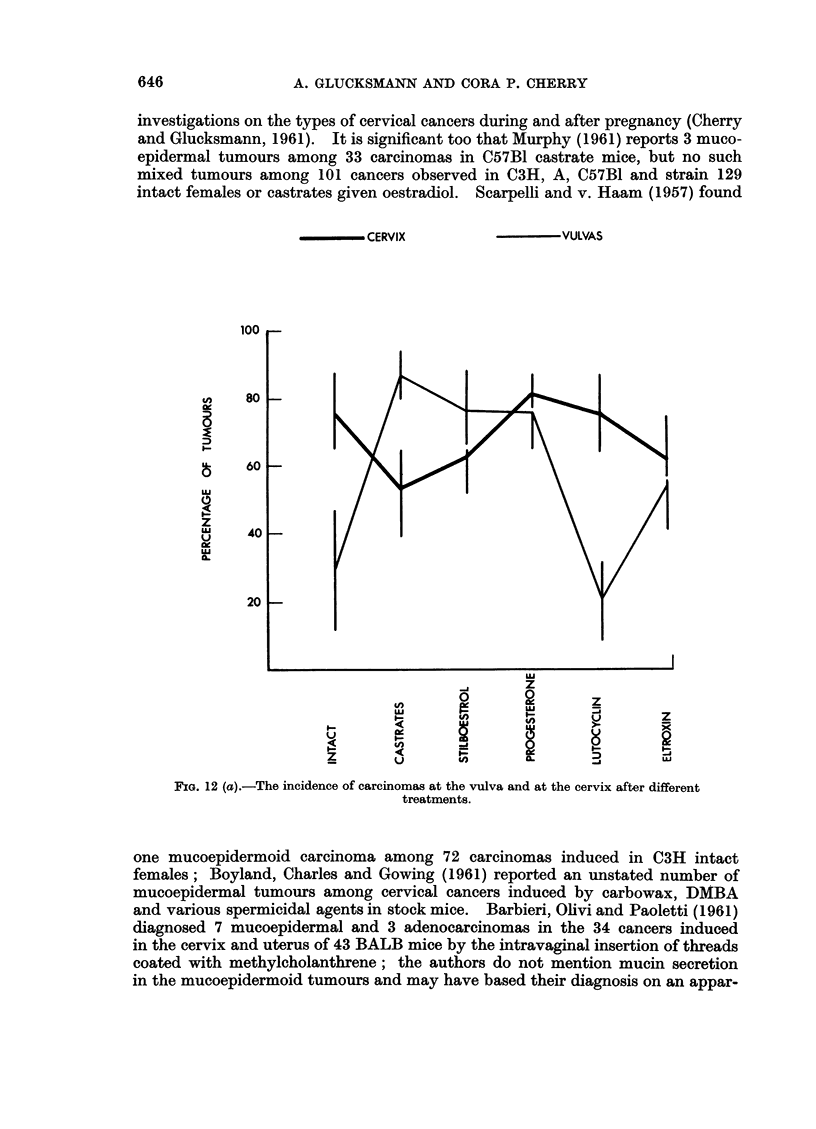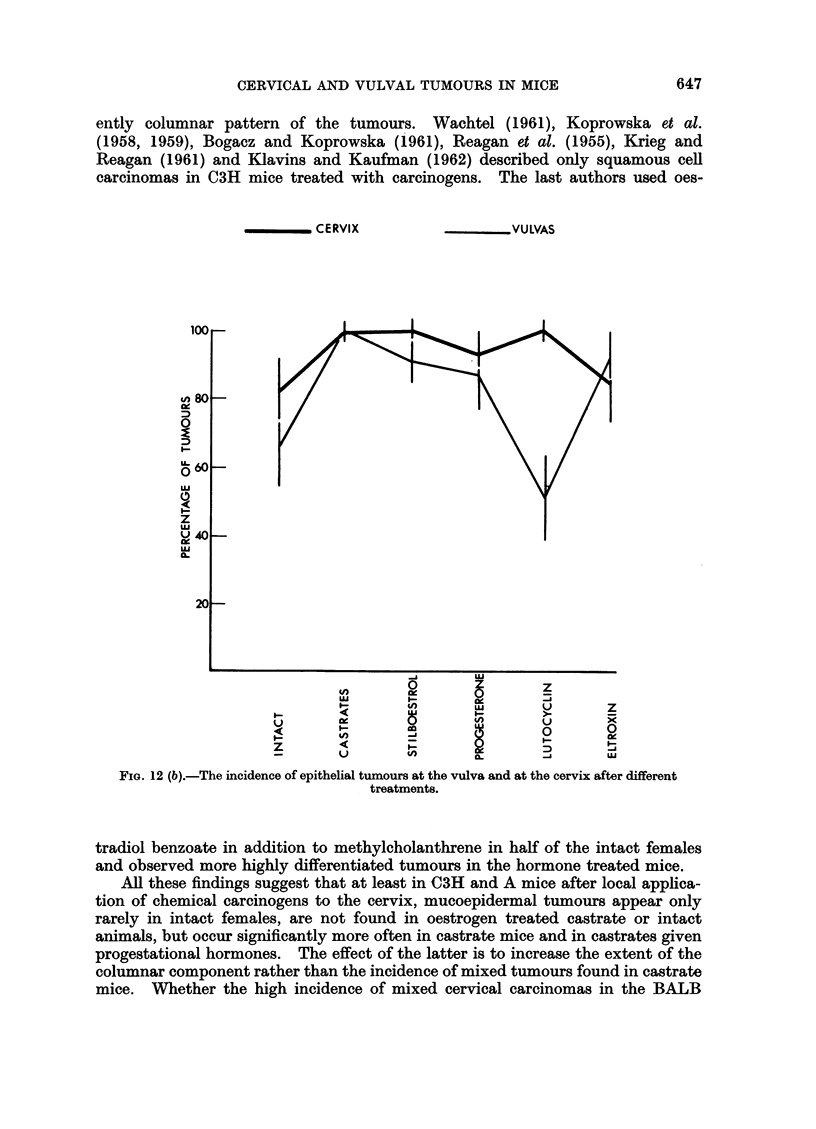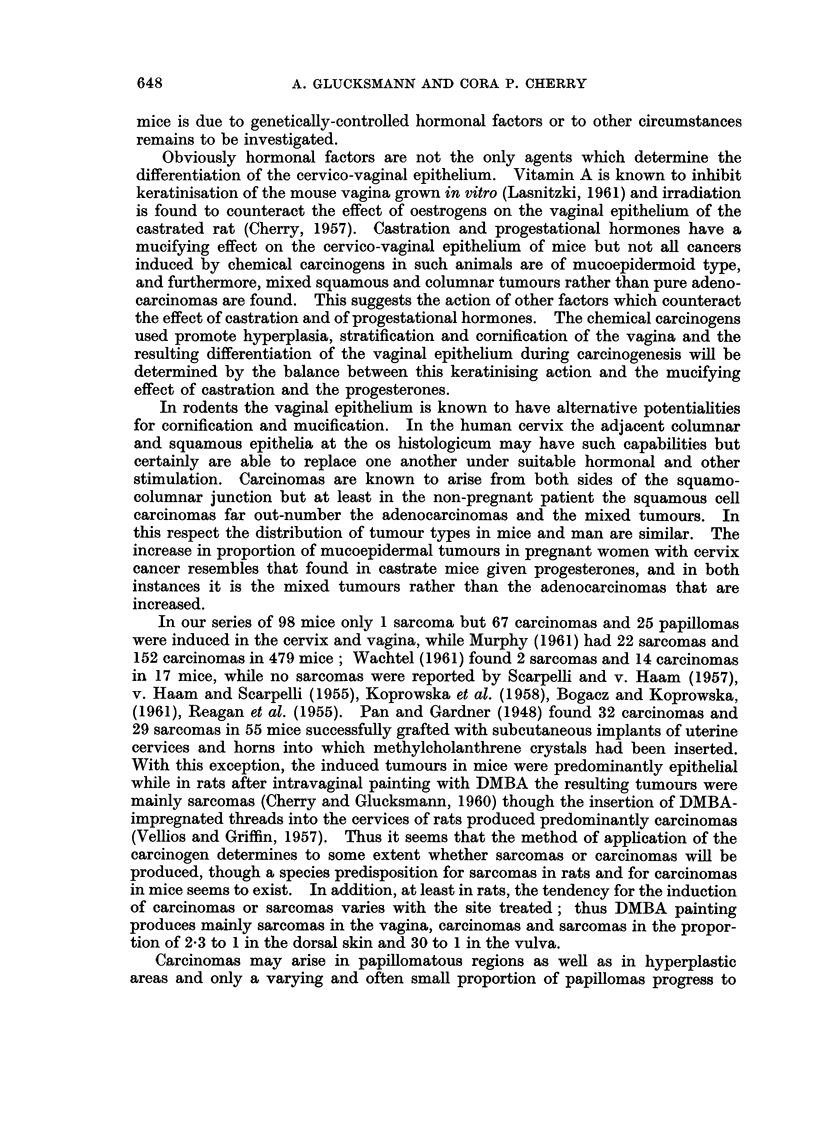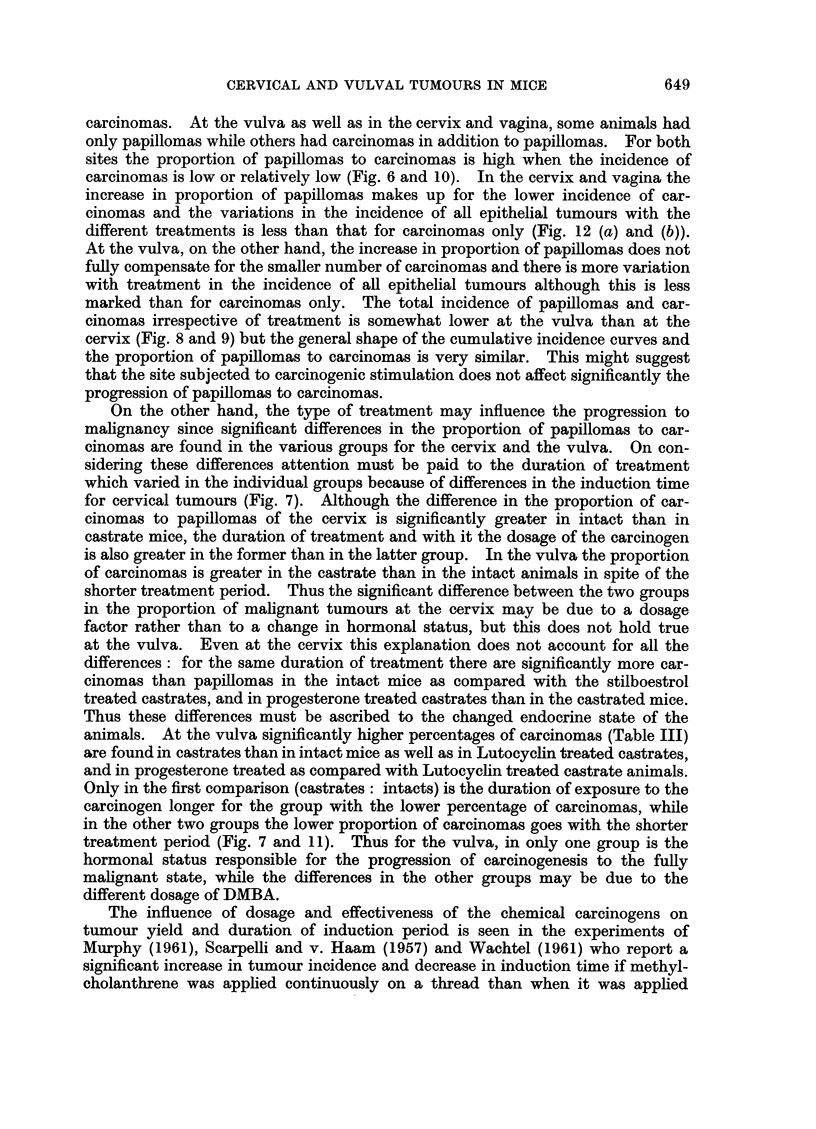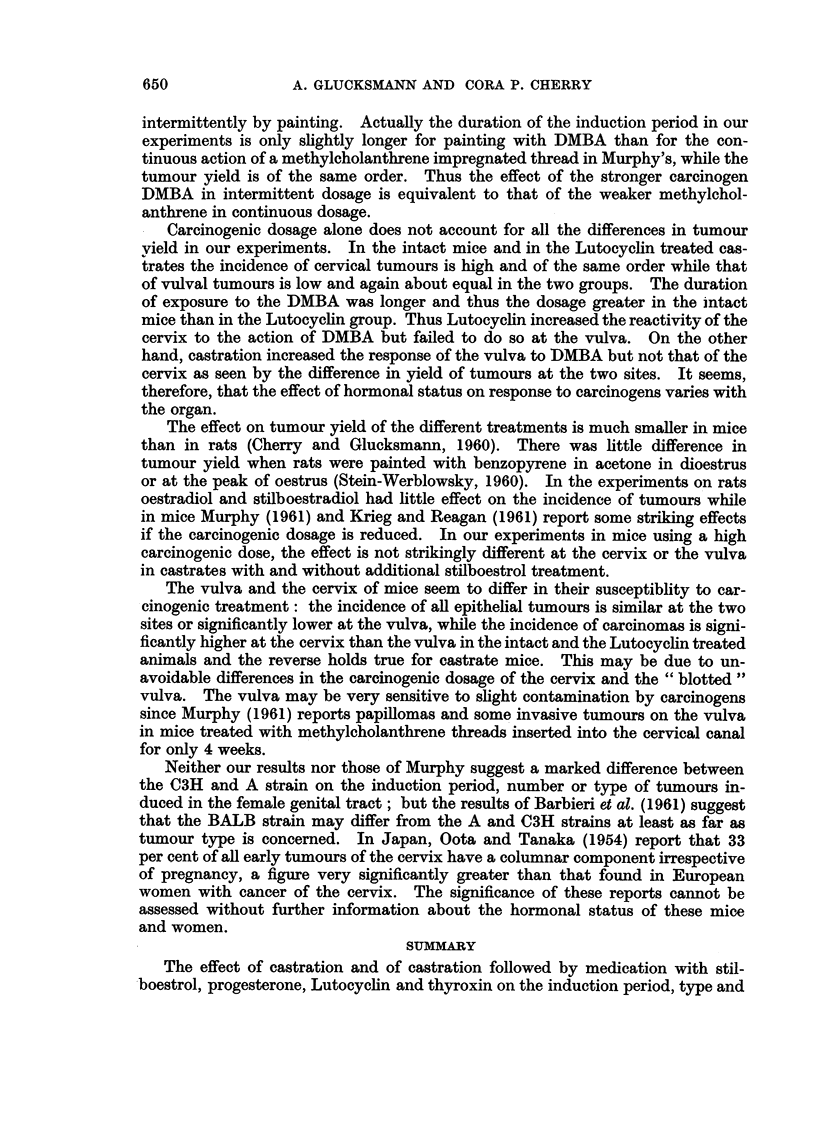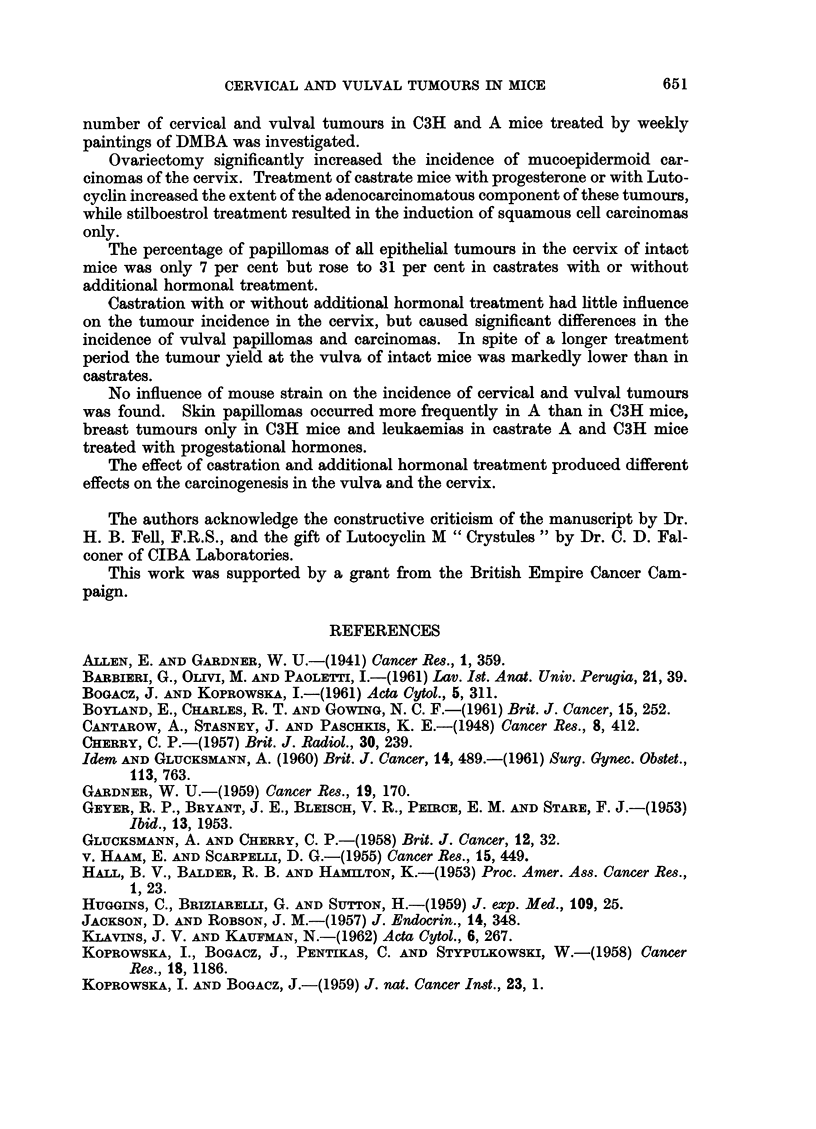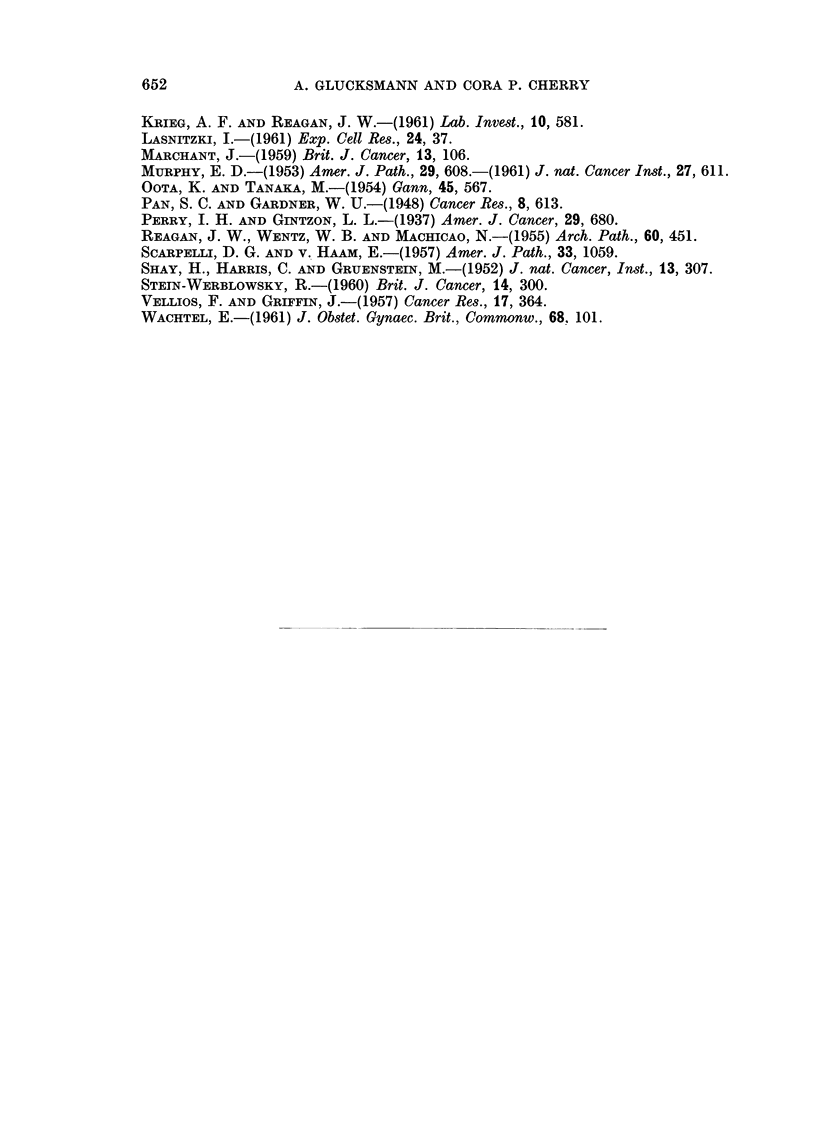# The Effect of Castration and of Additional Hormonal Treatments on the Induction of Cervical and Vulval Tumours in Mice

**DOI:** 10.1038/bjc.1962.75

**Published:** 1962-12

**Authors:** A. Glucksmann, Cora P. Cherry

## Abstract

**Images:**


### THE EFFECT OF CASTRATION AND OF ADDITIONAL HORMONAL TREATMENTS ON THE INDUCTION OF CERVICAL AND VULVAL TUMOURS IN MICE

A. GLUCKSMANN AND CORA P. CHERRY

*From the Strangeways Research Laboratory, Cambridge*

Received for publication August 17, 1962

A HIGH incidence of mixed cervical carcinomas, containing both a squamous and a columnar component, during and after pregnancy as compared with non-pregnant pre- and postmenopausal women (Cherry and Glucksmann, 1961) suggested that hormonal factors may influence the type of a tumour developing during a pregnancy. In order to put this hypothesis to an experimental test, the female genital tract of rats was treated with carcinogenic chemicals and the endocrine status of the animals was altered experimentally (Glucksmann and Cherry, 1958 ; Cherry and Glucksmann, 1960). The majority of the tumours induced in the rat were sarcomas and while the endocrine status did affect the induction time and the yield of tumours, the influence of the hormonal treatment on the resulting tumour type could not be ascertained since the tumours were sarcomas rather than carcinomas.

In mice carcinomas have been induced experimentally by the application of sex hormones (Allen and Gardner, 1941 ; Pan and Gardner, 1948 ; Gardner, 1959) and by the local application of chemical carcinogens of various types (Murphy, 1953, 1961 ; v. Haam and Scarpelli, 1955 ; Scarpelli and v. Haam, 1957 ; Reagan, Wentz and Machicao, 1955 ; Krieg and Reagan, 1961 ; Koprowska, Bogacz, Pentikas and Stypulkowski, 1958 ; Koprowska and Bogacz, 1959 ; Bogacz and Koprowska, 1961 ; Boyland, Charles and Gowing, 1961 ; Klavins and Kaufmann, 1962 ; Barbieri, Olivi and Paoletti, 1961 ; Wachtel, 1961). Most of the tumours were squamous cell carcinomas, though a few mucin-producing carcinomas were also found as well as a small number of sarcomas (Murphy, 1961). Some of these investigators have used hormonal changes as well as carcinogens and reported an effect of castration and of subsequent treatment with oestrogens on the induction time and yield of tumours (Murphy, 1961 ; Krieg and Reagan, 1961 ; Pan and Gardner, 1948 ; Jackson and Robson, 1957 ; Hall, Balder and Hamilton, 1953 ; Perry and Ginzton, 1937) but not on the type of the tumour. Similarly effects of castration and additional hormonal treatments have been reported for breast tumours in rats and mice (Huggins, Briziarelli and Sutton, 1959 ; Geyer, Bryant, Bleisch, Peirce and Stare, 1953 ; Cantarow, Stasney and Paschkis, 1948), the skin of mice (Marchant, 1959) and the vagina of rats (Cherry and Glucksmann, 1960 ; Glucksmann and Cherry, 1958). Shay, Harris and Gruenstein (1952) reported that following gastric installation of methylcholanthrene, castration and additional steroid application, mammary glandular tumours predominated in females and in males given oestradiol, spindle cell tumours and mesenteric sarcomas in males and in females given testosterone, while fibroadenomas of the breast occurred in castrated males and females. The present investigation was devised specifically to test whether the development of squamous and of columnar carcinomas could be influenced by hormonal factors.

### MATERIAL AND METHODS

Female C3H and Strong A mice 8–12 weeks old were used for the experiments. Intact and castrated animals were painted intravaginally once weekly with a 1 per cent solution in acetone of 9, 10-dimethyl-1,2-benzanthracene (DMBA) obtained from Messrs. L. Light & Co. The vagina was stretched open by dorsal flexion of the tail, a cotton wool swab on the end of a thin wire was inserted and the cervix and vagina painted by means of a rotary motion. In all the experiments the vulva was blotted with filter paper immediately after the painting to reduce contamination by the carcinogen.

Bilateral ovariectomy was performed under ether or nembutal anaesthesia on mice 4–8 weeks old, and the painting was started 1–4 weeks later. Groups of castrated mice received additional hormone treatment which was started a few days before the first vaginal painting.

The various experimental procedures are given in Table I in which only mice surviving for at least 75 days after the beginning of DMBA treatment are considered as " at risk ".

Stilboestrol was used for oestrogenic stimulation and added daily in a concentration of 20 $\mu$g. to the drinking bottles containing 200 c.c. of water. This dose was sufficient to restore the volume and cellularity of the atrophic castrate uterus to the state of the intact animal. In experiments on rats a similar concentration of stilboestrol was equivalent in its effect to the intramuscular injection of $2 \times 1$ $\mu$g. of oestradiol monobenzoate per week. The implantation of oestradiol-cholesterol pellets was avoided since it caused pyometra in the rats and death from purulent endometritis. Furthermore, in the control experiments the implantation of cholesterol pellets was found to influence the induction period and yield of tumours in rats and for this reason the simple method of oestrogenic stimulation by the addition of stilboestrol to the drinking water was chosen.

Progesterone (Organon Ltd.) was injected intramuscularly twice weekly in a dose of 0·2 mg. in one experiment while in another it was administered in the form of Lutocyclin M crystules (CIBA). The Lutocyclin was implanted subcutaneously in a dose of 50 mg. at the beginning of the experiment followed 2 months later by an implantation of 25 mg. and after a further 6 weeks by another dose of 25 mg.

L-Thyroxine sodium (Eltroxin, Glaxo) was added to the drinking water in a concentration of 0·01 mg. per 100 c.c. of water daily and in addition a dose of 3 $\mu$g. in Tyrode was injected intramuscularly once weekly.

Animals were killed when the presence of vaginal or cervical tumours was indicated by their prolapse or by bleeding on painting or when extensive vulval tumours or other conditions (mammary carcinoma or leukaemia) likely to cause death or suffering, required it. At autopsy, apart from other organs or tissues showing abnormalities, the uterus, cervix, vagina, vulval skin, adrenals and, in intact mice, the ovaries were fixed routinely in Zenker-acetic, dehydrated and embedded in paraffin and sectioned at 8 $\mu$. Except for large cervical and vaginal tumours, the halved blocks of cervicovaginal tissue were cut serially. Sections were stained with haematoxylin-eosin, by the periodic acid-Schiff technique (PAS) with or without previous diastase digestion, with Southgate's mucicarmine stain, with van Gieson's stain or with a modified " Azan " stain.

### RESULTS

### *Carcinoma induction in the cervix and vagina*

Intravaginal painting with DMBA of intact mice induced invasive carcinomas of the cervix or vagina in 13 of the 17 (76 per cent) animals at risk. These tumours usually involved the cervix and in many cases extended on to the vagina but in a few animals the growth was confined to the vagina. Only squamous cell carcinomas were found in the intact mice and the majority were well differentiated with good keratinisation and the formation of horn pearls (Fig. 1). A few of the tumours were more anaplastic and produced little keratinisation and few horn pearls.

Treatment with DMBA induced carcinomas of the cervix or vagina in 8 of the 15 (53 per cent) castrated animals at risk. Of these tumours 5 were of the squamous cell type while the remaining 3 were mixed carcinomas giving an incidence of 38 per cent of all cancers in this group (Table I). In these mixed tumours

TABLE I.—*Induction of Cervical Carcinomas in Mice by Local Application of DMBA*

| Treatment | Mice: Strain | Mice: Number at risk | Percentage of carcinomas | Percentage of mixed carcinomas of all carcinomas |
|---|---|---|---|---|
| Intact mice | C3H | 17 | 76 | 0 |
| Castrate mice | C3H | 15 | 53 | 38 |
| ,, + Stilboestrol | A | 21 | 62 | 0 |
| ,, + Progesterone | C3H | 16 | 81 | 31 |
| ,, + Lutocyclin | A | 16 | 75 | 42 |
| ,, + Eltroxin | C3H | 13 | 61 | 12 |

columnar mucin-producing as well as keratinising squamous components were present in separate foci or in the same tumour strand (Fig. 2–5). Both tumour strains showed definite malignant characteristics in their cytology and structure and could be distinguished easily from persisting normal mucin-secreting cells or glands surrounded by squamous celled carcinomas (Reagan *et al.*, 1955).

---

### EXPLANATION OF PLATE

FIG. 1.—A keratinising squamous cell carcinoma of the cervix of an intact C3H mouse induced by weekly paintings with DMBA over a period of 196 days. H. E. ×85.

FIG. 2.—A mucoepidermoid carcinoma of the cervix in a castrate C3H mouse induced by weekly paintings with DMBA over a period of 178 days (cf. also Fig. 3–5). At the endocervical end the tumour is predominantly adenocarcinomatous (cf. Fig. 4), followed by a region consisting of squamous cell carcinoma (cf. Fig. 3) which in turn is followed by solid formations of mucin-secreting tumour cells (cf. Fig. 5). P.A.S. ×14.

FIG. 3.—Part of the keratinising squamous cell carcinoma in Fig. 2. P.A.S. ×110.

FIG. 4.—Part of the adenocarcinomatous formation in Fig. 2. The black regions represent mucin secretion in the glandular formations. P.A.S. ×150.

FIG. 5.—The mucoepidermoid portion of the tumour in Fig. 2 has cells of squamous shape arranged in epithelial strands but containing mucin which appears as the intensely black cytoplasmic regions. P.A.S. ×110.

1

3

2

5

4

Glucksmann and Cherry.

DMBA painting of castrated mice given stilboestrol per os induced carcinomas of the cervix or vagina in 13 of the 21 (62 per cent) animals at risk. As in the intact mice all these tumours were squamous cell carcinomas most of which were well differentiated.

DMBA painting of castrated mice given progesterone by intramuscular injection induced carcinomas of the cervix or vagina in 13 of 16 (81 per cent) animals, and when the hormone was given as Lutocyclin crystules in 12 of 16 (75 per cent) mice. In both groups mixed tumours as well as squamous cell cancers were found. With the intermittent hormonal stimulation 4 of the 13 tumours (31 per cent) were mixed carcinomas while with the continuously acting Lutocyclin crystules 5 of the 12 cancers (42 per cent) were of this type (Table I). These mixed tumours contained separate squamous (Fig. 3) and columnar foci (Fig. 4) as well as some areas in which the two cell strains were intermingled (Fig. 5). The adenocarcinomatous element was more extensive with additional progestational stimulation than in the mixed tumours of the castrate mice where they were found only after prolonged search in serial sections.

In the castrates given Eltroxin, painting with DMBA induced carcinomas of the cervix or vagina in 8 of the 13 (61 per cent) mice at risk. Of these tumours 7 were of the squamous cell type and 1 was a mixed carcinoma (Table I). One of the mice had a leiomyosarcoma extending from the cervix to the uterus as well as a squamous cell carcinoma of the cervix.

The incidence of mixed carcinomas as proportion of all cancers is given in Table I. The group of intact mice plus castrates given stilboestrol plus those given thyroxine had only 3 per cent of mixed tumours while the castrates with or without progestational treatment had 36 per cent, a statistically significant difference of 33 $\pm$ 8·73.

The total cervico-vaginal tumour incidence in the various groups did not differ significantly though there were slightly fewer carcinomas in the castrates without hormonal treatment. In most of the animals with carcinomas, papillomas were also observed while an additional group of mice had squamous papillomas but no carcinomas. The incidence of carcinomas and papillomas for the various treatment groups is given in Table II and in the histogram of Fig. 6 which shows

TABLE II.—*Incidence of Carcinomas and of All Epithelial Tumours at the Cervix*

| Treatment | Number at risk | Percentage of: Carcinomas | Percentage of: Epithelial tumours |
|---|---|---|---|
| DMBA | | | |
| Intact | 17 | 76$\pm$10·4 | 82$\pm$9·3 |
| Castrate | 15 | 53$\pm$12·9 | 100$\pm$2·6 |
| „ + Stilboestrol | 21 | 62$\pm$10·6 | 100$\pm$2·2 |
| „ + Progesterone | 16 | 81$\pm$9·8 | 94$\pm$5·9 |
| „ + Lutocyclin | 16 | 75$\pm$10·8 | 100$\pm$2·5 |
| „ + Eltroxin | 13 | 61$\pm$13·5 | 84$\pm$10·2 |

some variation in the proportion of carcinomas to papillomas with treatment. The percentage of papillomas in intact mice was only 7 per cent but rose to 31 per cent (difference 24 $\pm$ 8·6) in castrates with or without additional treatment. The proportion of carcinomas of all epithelial tumours (Table III) is significantly

FIG. 6.—Histogram showing the incidence of papillomas and carcinomas of the cervix after different additional hormonal treatments.

TABLE III.—*Proportion of Carcinomas of All Epithelial Tumours at the Cervix and Vulva*

| | Cervix | | Vulva | |
|---|---|---|---|---|
| Treatment | Number of tumours | % Carcinomas | Number of tumours | % Carcinomas |
| Intact | 14 | 93±6·8 | 11 | 45±15·0 |
| Castrate | 15 | 53±12·9 | 15 | 87±8·7 |
| ,, + Stilboestrol | 21 | 62±10·6 | 19 | 84±8·4 |
| ,, + Progesterone | 15 | 87±8·7 | 14 | 86±9·3 |
| ,, + Lutocyclin | 16 | 75±10·8 | 8 | 37±17·1 |
| ,, + Eltroxin | 11 | 73±13·4 | 12 | 58±14·2 |

greater in intact than in castrate mice (difference 40 ± 14·6), in intact than in stilboestrol-treated castrates (difference 31 ± 12·6) and in progesterone treated castrates than in castrated animals (difference 34 ± 15·5).

*Time ranges in tumour induction*

Since animals were killed only when there was definite clinical evidence of cervico-vaginal tumours or when large vulval growths or other conditions made it necessary, the true induction time could not be determined. The duration from the beginning of treatment to (*a*) the histological verification of the first carcinoma or papilloma, or (*b*) the time a 50 per cent incidence of tumours was reached can be assessed from Fig. 7 and Table IV ; the error in this time estimate is presumably similar in all treated groups. The variation in sensitivity of individual mice is reflected in the S-type curves of Fig. 7 and makes the interval to first

FIG. 7.—Cumulative percentage incidence of cervical carcinomas.

TABLE IV.—*Induction Period for Cervical Tumours in Mice*

| Treatment | Strain | Interval in days to appearance of: Carcinomas First | Carcinomas 50% | Epithelial tumours 50% |
|---|---|---|---|---|
| Intact mice . . . | C3H . | 168 | 205 | 200 |
| Castrate mice . . . | C3H . | 126 | 180 | 140 |
| „ + Stilboestrol . | A . | 85 | 201 | 175 |
| „ + Progesterone . | C3H . | 150 | 166 | 166 |
| „ + Lutocyclin . | A . | 98 | 135 | 128 |
| „ + Eltroxin . . | C3H . | 80 | 164 | 145 |

tumour appearance less reliable than the estimate of the induction time for a 50 per cent tumour incidence. Intact and stilboestrol treated castrate mice were slowest to reach the 50 per cent level while those treated with Lutocyclin were the fastest. A stilboestrol treated castrate mouse was one of the first to have a histologically confirmed carcinoma, but the interval between the first cancer and the 50 per cent level was the longest (116 days) while it was shortest for the

FIG. 8.—The induction of papillomas and carcinomas at the cervix.

progesterone treated animals (16 days). The strain of mice did not markedly affect the duration of the induction period and was of the same order for strain A and C3H mice.

Though in the same mouse papillomas may precede the appearance of carcinomas, in some mice papillomas may appear at the same time as carcinomas do in others, as Fig. 8 shows. The time for a 50 per cent incidence of all epithelial tumours (carcinomas plus papillomas) is given in the last column of Table IV and shows some shortening as compared with carcinoma induction for castrate mice, stilboestrol and Eltroxin treated castrates but no significant difference in the other 3 groups.

*Tumour induction in the vulva*

Although the vulva was blotted after painting to reduce the contamination by the carcinogen, the majority of the treated animals showed hyperplasia of the vulval epithelium which progressed to papillomas and carcinomas in a high proportion of mice. The papillomas tended to precede and to coexist with the carcinomas which were of the keratinising squamous cell type. Multiple carcin-

FIG. 9.—The induction of papillomas and carcinomas at the vulva.

omas and papillomas were found in a great number of vulvas in addition to hyperplastic regions.

Mice with vulval carcinomas were observed at the same time as others with papillomas (Fig. 9). While the cumulative percentage of mice with carcinomas rose steeply, that of mice with papillomas rose more slowly.

The incidence of mice with carcinomas and those with papillomas only is given in Table V and Fig. 10 ; there are significant differences between some of the groups. The incidence of carcinomas in intact mice was 29 per cent and in castrates 87 per cent (difference 58 $\pm$ 14·0) and of all epithelial tumours 65 per cent in intact and 100 per cent in castrate mice (difference 35 $\pm$ 11·8). This difference is very likely real since Fig. 7 and 11 indicate a longer induction period for cervical

TABLE V.—*Incidence of Carcinomas and of All Epithelial Tumours at the Vulva*

| Treatment | Number at risk | Percentage of Carcinomas | Percentage of Epithelial tumours |
|---|---|---|---|
| Intact . . . . . | 17 | 29±11·0 | 65±11·6 |
| Castrate . . . . | 15 | 87±8·7 | 100±2·6 |
| „ + Stilboestrol . . | 21 | 76±9·3 | 91±6·2 |
| „ + Progesterone . . | 16 | 75±10·8 | 87±8·4 |
| „ + Lutocyclin . . | 16 | 19±9·8 | 50±12·5 |
| „ + Eltroxin . . | 13 | 54±13·8 | 92±7·5 |

FIG. 10.—Histogram showing the incidence of papillomas and carcinomas of the vulva after different treatments.

tumours and thus a longer survival time for intact than for castrate mice. Thus in spite of more numerous exposures to DMBA over a longer period of time, the vulva of intact mice produced fewer tumours than that of the castrates. The difference in incidence of carcinomas (Table V) in intact animals and in castrates given stilboestrol is significant at the 95 per cent confidence level (difference

47 ± 14·4), but the difference in the incidence of all epithelial tumours in these two groups does not reach the same level of significance (difference 26 ± 13·1). The induction period for cervical tumours and the survival time for intact and stilboestrol treated castrates (Fig. 7 and 11) is very similar.

The progesterone treated castrates had significantly more carcinomas than the Lutocyclin treated group (difference 56 ± 14·6) and the difference in the incidence

FIG. 11.—Cumulative percentage incidence of vulval carcinomas.

of all epithelial tumours (difference 37 ± 15·1), though smaller, is still significant. The induction period for cervical tumours and with it the survival time was shorter in the Lutocyclin than in the progesterone treated mice (Fig. 7 and 11) and only the longest survivors in the former group produced vulval tumours. The difference in vulval tumour incidence between these animals may thus be due to the longer exposure to the carcinogen ; it may also be influenced by genetic differences in mouse strain.

The proportion of carcinomas of all epithelial tumours (Table III, Fig. 10) is significantly greater in castrate than in intact mice (difference 42 ± 17·3) which is the reverse of that found for the cervix. The proportion of carcinomas is significantly lower in the Lutocyclin treated group than in the castrates and in the progesterone treated animals (difference 50 ± 19·1 and 49 ± 19·4 respectively).

A comparison of Fig. 7 and Fig. 11 shows less tendency for an S-type of curve in the cumulative percentage of carcinomas at the vulva than for those at the cervix. In particular the initial slope of the curve is much steeper at the vulva than at the cervix.

*Remote tumours and influence of mouse strain on tumour induction*

In addition to tumours arising in the regions painted with DMBA, remote growths occurred in the breast, the skin and as leukaemias. Adenocarcinomas of cellular, papilliferous and cystic type were found in the breasts of C3H but not

TABLE VI.—*Incidence of Remote Tumours*

| Treatment | Mice: Strain | Mice: Number at risk | Skin: Carcinoma | Skin: Papilloma | Breast | Leukaemia |
|---|---|---|---|---|---|---|
| Intact mice | C3H | 17 | 0 | 0 | 6 | 0 |
| Castrate mice | C3H | 15 | 1 | 1 | 1 | 0 |
| „ + Stilboestrol | A | 21 | 0 | 9 | 0 | 0 |
| „ + Progesterone | C3H | 16 | 1 | 0 | 2 | 4 |
| „ + Lutocyclin | A | 16 | 1 | 3 | 0 | 1 |
| „ + Eltroxin | C3H | 13 | 1 | 6 | 3 | 0 |

of Strong A mice (Table VI). Among the C3H mice they appeared most frequently in the intact animals but this fact may be due to the longer induction period for cervix tumours and thus to the longer survival time in this group. The effect of castration on breast tumour induction is well known and the numbers in our experiments are too small to allow of any deductions about the effects of thyroxin and progesterone treatment.

Skin tumours were localised in the face, the mammary region from neck to groin, the abdomen, thorax, legs and tail. The majority were papillomas (Table VI) but 3 invasive carcinomas were found in 61 C3H and one in 37 Strong A mice. These figures do not suggest any variation with strain in the incidence of skin carcinomas. If, however, all 23 epithelial skin tumours are considered, the Strong A mice are seen to have a significantly greater incidence (35 per cent) than the C3H mice (15 per cent). The difference of 20 ± 9·1 is significant at the 95 per cent confidence level.

Leukaemias were found in 5 of 32 mice treated with progesterone or Lutocyclin but in none of the 66 mice treated in other ways. The significance of this observation needs further investigation.

While breast tumours occurred in C3H mice only, skin tumours more frequently in Strong A than in C3H mice, leukaemias seemed to be dependent on the treatment rather than on the strain of mice. It is thus relevant to analyse how far the results reported for the direct painting of the cervico-vaginal region and the vulva in mice given additional systemic treatment are due to the treatments rather than differences in the strain of mice used.

No significant difference between the various treatment groups was found in the incidence of cervical carcinomas. Irrespective of treatment additional to the DMBA application the incidence of carcinomas at this site amounted to 69 per cent for 61 C3H mice and to 67 per cent for 37 of the Strong A strain. The incidence for all epithelial tumours was 90 and 100 per cent respectively in the two strains. The proportion of carcinomas among the epithelial tumours was 76 per cent for C3H and 67 per cent for Strong A mice and not significantly different.

In the vulva the incidence of carcinomas was significantly lower in intact mice and in the Lutocyclin group, the former of which were C3H and the latter Strong A mice. If both these groups are included, vulval carcinomas occurred in 60 per cent of the C3H and in 51 per cent of the Strong A mice. If the two groups are excluded the figures are 73 and 76 per cent respectively. The incidence of all epithelial vulval tumours was 85 per cent for all C3H and 73 per cent for all mice of the Strong A strain and changed to 93 and 91 per cent respectively after exclusion of the intact and the Lutocyclin groups. The proportion of carcinomas as percentage of all epithelial tumours at the vulva was 71 per cent for C3H and 70 per cent for Strong A animals and after exclusion of the two low incidence groups changed to 78 and 84 per cent respectively.

There is thus no good evidence that the strain of mice determines the tumour incidence in the vulva and cervix in the way it does that of breast cancers. In Fig. 12 (*a*) and (*b*) the incidence of carcinomas and of all epithelial tumours at the cervix and vulva are illustrated according to the experimental treatments. There is comparatively little variation with treatment group in the incidence of cervical epithelial tumours ; this variation increases if carcinomas only are considered but does not reach statistical significance. At the vulva too, the variation in the incidence of carcinomas is greater than that of epithelial tumours particularly for the intact C3H mice and for the Strong A Lutocyclin treated castrates.

There is no close correlation between the incidence of epithelial tumours at the cervix and those of the vulva for the same treatment. The tumour yield at the vulva is lower than that at the cervix, equals it or is even greater. The only significant difference in the incidence of epithelial tumours at the two sites is seen in the Lutocyclin treated group (Fig. 12 (*b*)). The differences in the yield of carcinomas at the cervix and the vulva are greater and significant for 3 groups : in intact mice and Lutocyclin treated castrates the cervical cancers are more frequent than the vulval (difference $47 \pm 15{\cdot}1$ and $56 \pm 14{\cdot}6$ respectively) while in castrates the vulval carcinomas are more numerous than the cervical (differences $34 \pm 15{\cdot}5$). While significantly more skin tumours are found in Strong A than in C3H mice, no significant differences between the two strains are seen in the incidence of tumours at the vulva which has essentially the same structure as the skin in other parts. Murphy (1961) found no significant differences in the tumour yield and induction period at the cervix for C3H and Strong A mice.

### DISCUSSION

The most significant result of these investigations is the observation that in castrate C3H and A mice with or without treatment with progestational hormones the incidence of mucoepidermal tumours is 36 per cent, while it is only 3 per cent in intact C3H mice and castrate A and C3H animals treated with stilboestrol or thyroxin (difference $33 \pm 8{\cdot}7$). This suggests that hormonal influences can determine the histological type of a developing tumour, as was inferrred from the investigations on the types of cervical cancers during and after pregnancy (Cherry and Glucksmann, 1961). It is significant too that Murphy (1961) reports 3 mucoepidermal tumours among 33 carcinomas in C57Bl castrate mice, but no such mixed tumours among 101 cancers observed in C3H, A, C57Bl and strain 129 intact females or castrates given oestradiol. Scarpelli and v. Haam (1957) found

FIG. 12 (*a*).—The incidence of carcinomas at the vulva and at the cervix after different treatments.

one mucoepidermoid carcinoma among 72 carcinomas induced in C3H intact females ; Boyland, Charles and Gowing (1961) reported an unstated number of mucoepidermal tumours among cervical cancers induced by carbowax, DMBA and various spermicidal agents in stock mice. Barbieri, Olivi and Paoletti (1961) diagnosed 7 mucoepidermal and 3 adenocarcinomas in the 34 cancers induced in the cervix and uterus of 43 BALB mice by the intravaginal insertion of threads coated with methylcholanthrene ; the authors do not mention mucin secretion in the mucoepidermoid tumours and may have based their diagnosis on an apparently columnar pattern of the tumours. Wachtel (1961), Koprowska *et al.* (1958, 1959), Bogacz and Koprowska (1961), Reagan *et al.* (1955), Krieg and Reagan (1961) and Klavins and Kaufman (1962) described only squamous cell carcinomas in C3H mice treated with carcinogens. The last authors used oes-

FIG. 12 (*b*).—The incidence of epithelial tumours at the vulva and at the cervix after different treatments.

tradiol benzoate in addition to methylcholanthrene in half of the intact females and observed more highly differentiated tumours in the hormone treated mice.

All these findings suggest that at least in C3H and A mice after local application of chemical carcinogens to the cervix, mucoepidermal tumours appear only rarely in intact females, are not found in oestrogen treated castrate or intact animals, but occur significantly more often in castrate mice and in castrates given progestational hormones. The effect of the latter is to increase the extent of the columnar component rather than the incidence of mixed tumours found in castrate mice. Whether the high incidence of mixed cervical carcinomas in the BALB

mice is due to genetically-controlled hormonal factors or to other circumstances remains to be investigated.

Obviously hormonal factors are not the only agents which determine the differentiation of the cervico-vaginal epithelium. Vitamin A is known to inhibit keratinisation of the mouse vagina grown *in vitro* (Lasnitzki, 1961) and irradiation is found to counteract the effect of oestrogens on the vaginal epithelium of the castrated rat (Cherry, 1957). Castration and progestational hormones have a mucifying effect on the cervico-vaginal epithelium of mice but not all cancers induced by chemical carcinogens in such animals are of mucoepidermoid type, and furthermore, mixed squamous and columnar tumours rather than pure adenocarcinomas are found. This suggests the action of other factors which counteract the effect of castration and of progestational hormones. The chemical carcinogens used promote hyperplasia, stratification and cornification of the vagina and the resulting differentiation of the vaginal epithelium during carcinogenesis will be determined by the balance between this keratinising action and the mucifying effect of castration and the progesterones.

In rodents the vaginal epithelium is known to have alternative potentialities for cornification and mucification. In the human cervix the adjacent columnar and squamous epithelia at the os histologicum may have such capabilities but certainly are able to replace one another under suitable hormonal and other stimulation. Carcinomas are known to arise from both sides of the squamo-columnar junction but at least in the non-pregnant patient the squamous cell carcinomas far out-number the adenocarcinomas and the mixed tumours. In this respect the distribution of tumour types in mice and man are similar. The increase in proportion of mucoepidermal tumours in pregnant women with cervix cancer resembles that found in castrate mice given progesterones, and in both instances it is the mixed tumours rather than the adenocarcinomas that are increased.

In our series of 98 mice only 1 sarcoma but 67 carcinomas and 25 papillomas were induced in the cervix and vagina, while Murphy (1961) had 22 sarcomas and 152 carcinomas in 479 mice ; Wachtel (1961) found 2 sarcomas and 14 carcinomas in 17 mice, while no sarcomas were reported by Scarpelli and v. Haam (1957), v. Haam and Scarpelli (1955), Koprowska *et al.* (1958), Bogacz and Koprowska, (1961), Reagan *et al.* (1955). Pan and Gardner (1948) found 32 carcinomas and 29 sarcomas in 55 mice successfully grafted with subcutaneous implants of uterine cervices and horns into which methylcholanthrene crystals had been inserted. With this exception, the induced tumours in mice were predominantly epithelial while in rats after intravaginal painting with DMBA the resulting tumours were mainly sarcomas (Cherry and Glucksmann, 1960) though the insertion of DMBA-impregnated threads into the cervices of rats produced predominantly carcinomas (Vellios and Griffin, 1957). Thus it seems that the method of application of the carcinogen determines to some extent whether sarcomas or carcinomas will be produced, though a species predisposition for sarcomas in rats and for carcinomas in mice seems to exist. In addition, at least in rats, the tendency for the induction of carcinomas or sarcomas varies with the site treated ; thus DMBA painting produces mainly sarcomas in the vagina, carcinomas and sarcomas in the proportion of 2·3 to 1 in the dorsal skin and 30 to 1 in the vulva.

Carcinomas may arise in papillomatous regions as well as in hyperplastic areas and only a varying and often small proportion of papillomas progress to carcinomas. At the vulva as well as in the cervix and vagina, some animals had only papillomas while others had carcinomas in addition to papillomas. For both sites the proportion of papillomas to carcinomas is high when the incidence of carcinomas is low or relatively low (Fig. 6 and 10). In the cervix and vagina the increase in proportion of papillomas makes up for the lower incidence of carcinomas and the variations in the incidence of all epithelial tumours with the different treatments is less than that for carcinomas only (Fig. 12 (*a*) and (*b*)). At the vulva, on the other hand, the increase in proportion of papillomas does not fully compensate for the smaller number of carcinomas and there is more variation with treatment in the incidence of all epithelial tumours although this is less marked than for carcinomas only. The total incidence of papillomas and carcinomas irrespective of treatment is somewhat lower at the vulva than at the cervix (Fig. 8 and 9) but the general shape of the cumulative incidence curves and the proportion of papillomas to carcinomas is very similar. This might suggest that the site subjected to carcinogenic stimulation does not affect significantly the progression of papillomas to carcinomas.

On the other hand, the type of treatment may influence the progression to malignancy since significant differences in the proportion of papillomas to carcinomas are found in the various groups for the cervix and the vulva. On considering these differences attention must be paid to the duration of treatment which varied in the individual groups because of differences in the induction time for cervical tumours (Fig. 7). Although the difference in the proportion of carcinomas to papillomas of the cervix is significantly greater in intact than in castrate mice, the duration of treatment and with it the dosage of the carcinogen is also greater in the former than in the latter group. In the vulva the proportion of carcinomas is greater in the castrate than in the intact animals in spite of the shorter treatment period. Thus the significant difference between the two groups in the proportion of malignant tumours at the cervix may be due to a dosage factor rather than to a change in hormonal status, but this does not hold true at the vulva. Even at the cervix this explanation does not account for all the differences : for the same duration of treatment there are significantly more carcinomas than papillomas in the intact mice as compared with the stilboestrol treated castrates, and in progesterone treated castrates than in the castrated mice. Thus these differences must be ascribed to the changed endocrine state of the animals. At the vulva significantly higher percentages of carcinomas (Table III) are found in castrates than in intact mice as well as in Lutocyclin treated castrates, and in progesterone treated as compared with Lutocyclin treated castrate animals. Only in the first comparison (castrates : intacts) is the duration of exposure to the carcinogen longer for the group with the lower percentage of carcinomas, while in the other two groups the lower proportion of carcinomas goes with the shorter treatment period (Fig. 7 and 11). Thus for the vulva, in only one group is the hormonal status responsible for the progression of carcinogenesis to the fully malignant state, while the differences in the other groups may be due to the different dosage of DMBA.

The influence of dosage and effectiveness of the chemical carcinogens on tumour yield and duration of induction period is seen in the experiments of Murphy (1961), Scarpelli and v. Haam (1957) and Wachtel (1961) who report a significant increase in tumour incidence and decrease in induction time if methylcholanthrene was applied continuously on a thread than when it was applied intermittently by painting. Actually the duration of the induction period in our experiments is only slightly longer for painting with DMBA than for the continuous action of a methylcholanthrene impregnated thread in Murphy's, while the tumour yield is of the same order. Thus the effect of the stronger carcinogen DMBA in intermittent dosage is equivalent to that of the weaker methylcholanthrene in continuous dosage.

Carcinogenic dosage alone does not account for all the differences in tumour yield in our experiments. In the intact mice and in the Lutocyclin treated castrates the incidence of cervical tumours is high and of the same order while that of vulval tumours is low and again about equal in the two groups. The duration of exposure to the DMBA was longer and thus the dosage greater in the intact mice than in the Lutocyclin group. Thus Lutocyclin increased the reactivity of the cervix to the action of DMBA but failed to do so at the vulva. On the other hand, castration increased the response of the vulva to DMBA but not that of the cervix as seen by the difference in yield of tumours at the two sites. It seems, therefore, that the effect of hormonal status on response to carcinogens varies with the organ.

The effect on tumour yield of the different treatments is much smaller in mice than in rats (Cherry and Glucksmann, 1960). There was little difference in tumour yield when rats were painted with benzopyrene in acetone in dioestrus or at the peak of oestrus (Stein-Werblowsky, 1960). In the experiments on rats oestradiol and stilboestradiol had little effect on the incidence of tumours while in mice Murphy (1961) and Krieg and Reagan (1961) report some striking effects if the carcinogenic dosage is reduced. In our experiments in mice using a high carcinogenic dose, the effect is not strikingly different at the cervix or the vulva in castrates with and without additional stilboestrol treatment.

The vulva and the cervix of mice seem to differ in their susceptiblity to carcinogenic treatment : the incidence of all epithelial tumours is similar at the two sites or significantly lower at the vulva, while the incidence of carcinomas is significantly higher at the cervix than the vulva in the intact and the Lutocyclin treated animals and the reverse holds true for castrate mice. This may be due to unavoidable differences in the carcinogenic dosage of the cervix and the " blotted " vulva. The vulva may be very sensitive to slight contamination by carcinogens since Murphy (1961) reports papillomas and some invasive tumours on the vulva in mice treated with methylcholanthrene threads inserted into the cervical canal for only 4 weeks.

Neither our results nor those of Murphy suggest a marked difference between the C3H and A strain on the induction period, number or type of tumours induced in the female genital tract ; but the results of Barbieri *et al.* (1961) suggest that the BALB strain may differ from the A and C3H strains at least as far as tumour type is concerned. In Japan, Oota and Tanaka (1954) report that 33 per cent of all early tumours of the cervix have a columnar component irrespective of pregnancy, a figure very significantly greater than that found in European women with cancer of the cervix. The significance of these reports cannot be assessed without further information about the hormonal status of these mice and women.

### SUMMARY

The effect of castration and of castration followed by medication with stilboestrol, progesterone, Lutocyclin and thyroxin on the induction period, type and number of cervical and vulval tumours in C3H and A mice treated by weekly paintings of DMBA was investigated.

Ovariectomy significantly increased the incidence of mucoepidermoid carcinomas of the cervix. Treatment of castrate mice with progesterone or with Lutocyclin increased the extent of the adenocarcinomatous component of these tumours, while stilboestrol treatment resulted in the induction of squamous cell carcinomas only.

The percentage of papillomas of all epithelial tumours in the cervix of intact mice was only 7 per cent but rose to 31 per cent in castrates with or without additional hormonal treatment.

Castration with or without additional hormonal treatment had little influence on the tumour incidence in the cervix, but caused significant differences in the incidence of vulval papillomas and carcinomas. In spite of a longer treatment period the tumour yield at the vulva of intact mice was markedly lower than in castrates.

No influence of mouse strain on the incidence of cervical and vulval tumours was found. Skin papillomas occurred more frequently in A than in C3H mice, breast tumours only in C3H mice and leukaemias in castrate A and C3H mice treated with progestational hormones.

The effect of castration and additional hormonal treatment produced different effects on the carcinogenesis in the vulva and the cervix.

The authors acknowledge the constructive criticism of the manuscript by Dr. H. B. Fell, F.R.S., and the gift of Lutocyclin M " Crystules " by Dr. C. D. Falconer of CIBA Laboratories.

This work was supported by a grant from the British Empire Cancer Campaign.

### REFERENCES

ALLEN, E. AND GARDNER, W. U.—(1941) *Cancer Res.*, **1**, 359.

BARBIERI, G., OLIVI, M. AND PAOLETTI, I.—(1961) *Lav. Ist. Anat. Univ. Perugia*, **21**, 39.

BOGACZ, J. AND KOPROWSKA, I.—(1961) *Acta Cytol.*, **5**, 311.

BOYLAND, E., CHARLES, R. T. AND GOWING, N. C. F.—(1961) *Brit. J. Cancer*, **15**, 252.

CANTAROW, A., STASNEY, J. AND PASCHKIS, K. E.—(1948) *Cancer Res.*, **8**, 412.

CHERRY, C. P.—(1957) *Brit. J. Radiol.*, **30**, 239.

*Idem* AND GLUCKSMANN, A. (1960) *Brit. J. Cancer*, **14**, 489.—(1961) *Surg. Gynec. Obstet.*, **113**, 763.

GARDNER, W. U.—(1959) *Cancer Res.*, **19**, 170.

GEYER, R. P., BRYANT, J. E., BLEISCH, V. R., PEIRCE, E. M. AND STARE, F. J.—(1953) *Ibid.*, **13**, 1953.

GLUCKSMANN, A. AND CHERRY, C. P.—(1958) *Brit. J. Cancer*, **12**, 32.

v. HAAM, E. AND SCARPELLI, D. G.—(1955) *Cancer Res.*, **15**, 449.

HALL, B. V., BALDER, R. B. AND HAMILTON, K.—(1953) *Proc. Amer. Ass. Cancer Res.*, **1**, 23.

HUGGINS, C., BRIZIARELLI, G. AND SUTTON, H.—(1959) *J. exp. Med.*, **109**, 25.

JACKSON, D. AND ROBSON, J. M.—(1957) *J. Endocrin.*, **14**, 348.

KLAVINS, J. V. AND KAUFMAN, N.—(1962) *Acta Cytol.*, **6**, 267.

KOPROWSKA, I., BOGACZ, J., PENTIKAS, C. AND STYPULKOWSKI, W.—(1958) *Cancer Res.*, **18**, 1186.

KOPROWSKA, I. AND BOGACZ, J.—(1959) *J. nat. Cancer Inst.*, **23**, 1.

Krieg, A. F. and Reagan, J. W.—(1961) *Lab. Invest.*, **10**, 581.
Lasnitzki, I.—(1961) *Exp. Cell Res.*, **24**, 37.
Marchant, J.—(1959) *Brit. J. Cancer*, **13**, 106.
Murphy, E. D.—(1953) *Amer. J. Path.*, **29**, 608.—(1961) *J. nat. Cancer Inst.*, **27**, 611.
Oota, K. and Tanaka, M.—(1954) *Gann*, **45**, 567.
Pan, S. C. and Gardner, W. U.—(1948) *Cancer Res.*, **8**, 613.
Perry, I. H. and Gintzon, L. L.—(1937) *Amer. J. Cancer*, **29**, 680.
Reagan, J. W., Wentz, W. B. and Machicao, N.—(1955) *Arch. Path.*, **60**, 451.
Scarpelli, D. G. and v. Haam, E.—(1957) *Amer. J. Path.*, **33**, 1059.
Shay, H., Harris, C. and Gruenstein, M.—(1952) *J. nat. Cancer, Inst.*, **13**, 307.
Stein-Werblowsky, R.—(1960) *Brit. J. Cancer*, **14**, 300.
Vellios, F. and Griffin, J.—(1957) *Cancer Res.*, **17**, 364.
Wachtel, E.—(1961) *J. Obstet. Gynaec. Brit., Commonw.*, **68**, 101.